# Gene Analysis of Four Families with Severe Peripartum Cardiomyopathy Reveals Known Gene Mutations and Supports the Recent Call for Screening

**DOI:** 10.31083/j.rcm2511399

**Published:** 2024-11-14

**Authors:** Mengmeng Li, Kaili Yin, Liang Chen, Jiazhen Chang, Na Hao

**Affiliations:** ^1^Department of Obstetrics & Gynecology, National Clinical Research Center for Obstetric & Gynecologic Diseases, Peking Union Medical College Hospital, Chinese Academy of Medical Science and Peking Union Medical College, 100730 Beijing, China; ^2^State Key Laboratory of Cardiovascular Disease, Fuwai Hospital, National Center for Cardiovascular Diseases, Chinese Academy of Medical Sciences and Peking Union Medical College, 100037 Beijing, China

**Keywords:** peripartum cardiomyopathy, genetics, pregnancy, genetic counselling

## Abstract

**Background:**

Peripartum cardiomyopathy (PPCM) is a rare disease that causes maternal morbidity and mortality worldwide. However, the etiology of PPCM is still unclear, and the rate of recovery varies between patients. Understanding the genetic factors underpinning PPCM may provide new insights into its pathogenesis.

**Methods:**

This genetic study screened six patients with severe PPCM and their family members using a panel of 204 genes associated with inherited cardiomyopathy.

**Results:**

The six probands progressed to severe cardiac dysfunction during follow-up, with a low left ventricular ejection fraction of <30% and a significant increase in left ventricular end-diastolic diameter. Genetic analysis showed that four of the six probands had pathogenic mutations. No specific mutation was identified in the other two probands. Further screening of the probands’ families identified that eight family members shared the same mutation with their probands. The total positive genetic mutation rate was 46% (12/26). Among those with genetic mutations, women who had pregnancies showed symptoms of heart disease.

**Conclusions:**

For PPCM patients with a genetic predisposition, pregnancy may exert pathogenic effects in terms of disease initiation and progression. Patients with PPCM with a first-degree relative diagnosed with inherited cardiomyopathy may benefit from genetic counselling.

## 1. Introduction 

Peripartum cardiomyopathy (PPCM) is a rare complication of pregnancy [1]. It is characterized by unexplained left ventricular (LV) systolic dysfunction and the development of heart failure (HF) occurring late in the 3rd trimester [2, 3]. The incidence of PPCM has been reported to be between 1 in 1000 to 1 in 4000 live births depending on geographic location [4, 5]. Notably, PPCM is more common in women of African descent, such as women from Haiti and Nigeria [6]. Aside from the Black race factor, advanced maternal age, multiple gestation, pregnancy-associated hypertension, and prolonged tocolysis have also been identified as strong risk factors for PPCM [7]. The outcomes of PPCM are highly heterogeneous, ranging from complete recovery of LV function to persistent dilated cardiomyopathy (DCM) and end-stage HF [8]. The prognosis of PPCM is dependent on the recovery from HF during the first 6 months postpartum, with mortality rates ranging from 2% in Germany to 12.6% in South Africa [9, 10].

The mechanisms underpinning the pathogenesis of PPCM are multifactorial and poorly understood. Hypotheses elucidating the underlying mechanisms include pathological responses to the hemodynamic changes of pregnancy, viral myocarditis, inflammation, abnormal autoimmune responses, and genetic predisposition [11, 12, 13]. Hemodynamic stress during pregnancy, which significantly increases cardiac workload, has often been proposed as the cause of PPCM [11]. However, hemodynamic shifts occur between the 1st and 2nd trimesters, when most women develop HF [14]. In contrast, patients with PPCM often present with symptoms during the 3rd trimester or after delivery [15]. Thus, hemodynamic stress is not likely to be a significant etiology of PPCM. Induction of antiangiogenic factors is also a possible factor leading to PPCM [16]. Particularly, 16-kDa prolactin derived from oxidative stress-mediated cleavage of hormone prolactin, may play a role in driving PPCM by causing endothelial damage [17]. A theory named the “two-hit” mechanism is thought to be crucial in PPCM development [18]. An underlying genetic mutation and imbalances in cardiac stressors mediated by vascular and hormonal actions are the basis of the two-hit mechanism [18, 19].

PPCM can be classified as an initial manifestation of familial dilated cardiomyopathy (FDCM) associated with pregnancy [20]. These conditions share some clinical features, including decreased systolic function and cardiac enlargement. In general, a high recovery rate has been reported for patients with PPCM after treatment, but major events and persistent severe cardiomyopathy are also observed, with a mortality rate of around 10% [21]. In these severe cases, patients with significant LV systolic dysfunction who have undergone heart transplantation account for at least 15% of patients with PPCM [1]. Notably, it has long been observed that PPCM may have a genetic component. The genetic similarities between PPCM and DCM imply a genetic etiology, and familial clustering of PPCM has been reported [20, 22]. We speculate that a genetic predisposition toward cardiomyopathy and HF may lead to more severe disease progression.

Currently, there are no clinical genetic testing recommendations for PPCM, whereas testing for DCM is widely performed [23, 24]. In this study, we conducted genetic mutation screening for individuals with end-stage PPCM and their family members by screening a panel of 204 genes associated with inherited cardiomyopathy to identify the gene mutations. We aimed to investigate the impact of cardiomyopathy-associated mutations on the development of severe PPCM and the influence of pregnancy on the progression of heart disease.

## 2. Materials and Methods

### 2.1 Patient Population

Patients with PPCM were enrolled based on the presence of HF secondary to LV dysfunction occurring in the last month of pregnancy or in the 5 months after delivery with no other cause of HF [25]. We included six patients with end-stage PPCM from six independent families who developed severe HF that gradually led to early death or heart transplantation. The clinical data of each patient and their relatives were collected. This study was approved by the ethics committee of our hospital. All participants provided written informed consent.

### 2.2 Genetic Screening 

Genetic testing of these patients was performed as reported previously [26]. Genomic DNA was extracted from peripheral blood samples obtained from six patients with PPCM and their relatives using the QIAamp DNA Blood Mini Kit (Qiagen, Hilden, Germany) according to the manufacturer’s protocol. The probands were screened for 204 genes (**Supplementary Table 1**) known to be associated with inherited cardiomyopathy via SeqCap EZ MedExome Panel (Roche NimbleGen, Basel, Switzerland) using the Illumina 2500 platform (Illumina, San Diego, CA, USA). All exonic regions of these panel genes, along with a 20-base-pair flanking intronic zone, were targeted for sequencing. The mean sequencing depth was 250×, and >98% of the targeted zone was covered with >20 reads.

All identified variants with mean allele frequencies of <0.5% in the control population of the ExAC, gnomAD (http://gnomad.broadinstitute.org/), 1000 Genomes Project (http://www.1000genomes.org) were processed for pathogenicity evaluation. The pathogenesis of the mutations was evaluated according to the American College of Medical Genetics and Genomics (ACMG) standards and guidelines [27]. Variants defined as pathogenic or likely pathogenic were obtained for this study. All candidate pathogenic mutations were verified by Sanger sequencing.

### 2.3 Histopathology Examination

Myocardial biopsy specimens were fixed in 10% formalin solution for subsequent histological analysis. Sections of these tissue specimens were then subjected to Masson’s trichrome staining.

### 2.4 Family Screening

The probands’ relatives underwent cardiac ultrasound echocardiography, electrocardiogram (ECG), and clinical inquiries about their infertility history and medical history. If pathogenic mutations were identified in the probands, the corresponding mutation was examined among the proband’s relatives by Sanger sequencing.

## 3. Results

### 3.1 Clinical Characteristics and Follow-up

Relatives of the six Probands were recruited and their clinical data are summarized in Table 1. The mean ± standard deviation age at diagnosis was 28.6 ± 2.3 SD years and ranged from 26 to 32 years. Two women were diagnosed in their 3rd trimester, while four women were diagnosed postpartum.

**Table 1.** **Clinical data of the six probands with PPCM**.

Case	P1	P2	P3	P4	P5	P6
Age, (yrs)	26	26	29	29	30	32
BMI	22.66	20.55	18.22	18.33	23.44	14.77
Timing at diagnosis	2–3 days postpartum	3rd trimester	3rd trimester	6 hours postpartum	3 days postpartum	3 months postpartum
Pregnancy	1	2	1	1	1	1
ECG/Arrhythmia	AF	VPBs	VPBs	PSVT	VPBs	NSVT
LVEF (%)	29	19	19	25	25	24
LVEDD (cm)	6.1	7.4	6.2	7.3	6.4	8.5
HR, beats/min	60	60	80	72	82	75
SBP, mmHg	99	90	80	94	101	92
DBP, mmHg	67	60	70	65	77	53
Taking ACEi/ARB	ACEi/ARB	ACEi/ARB	ACEi/ARB	ACEi/ARB	ACEi/ARB	ACEi/ARB
Taking BB	Beta-blocker	Beta-blocker	Beta-blocker	Beta-blocker	Beta-blocker	Beta-blocker
Diabetes	Diabetes	N	N	N	N	N
Hypertension	N	N	N	N	Hypertension	Hypertension
Smoking	N	N	N	N	N	N
Alcoholism	N	N	N	N	N	N
Preeclampsia	N	N	N	N	N	N
Family history	Y	Y	N	N	N	N

BMI, body mass index; P1, proband 1; LVEF, left ventricular ejection fraction; LVEDD, left ventricular diastolic diameter; VPBs, ventricular premature beats; NSVT, non-sustained ventricular tachycardia; AF, atrial fibrillation; PSVT, paroxysmal supraventricular tachycardia; HR, heart rate; SBP, systolic blood pressure; DBP; diastolic blood pressure; ACEi, angiotensin-converting enzyme inhibitor; ARB, angiotensin receptor blocker; BB, beta-blocker; PPCM, peripartum cardiomyopathy; ECG, electrocardiogram; N, no; Y, yes.

**Fig. 1.**
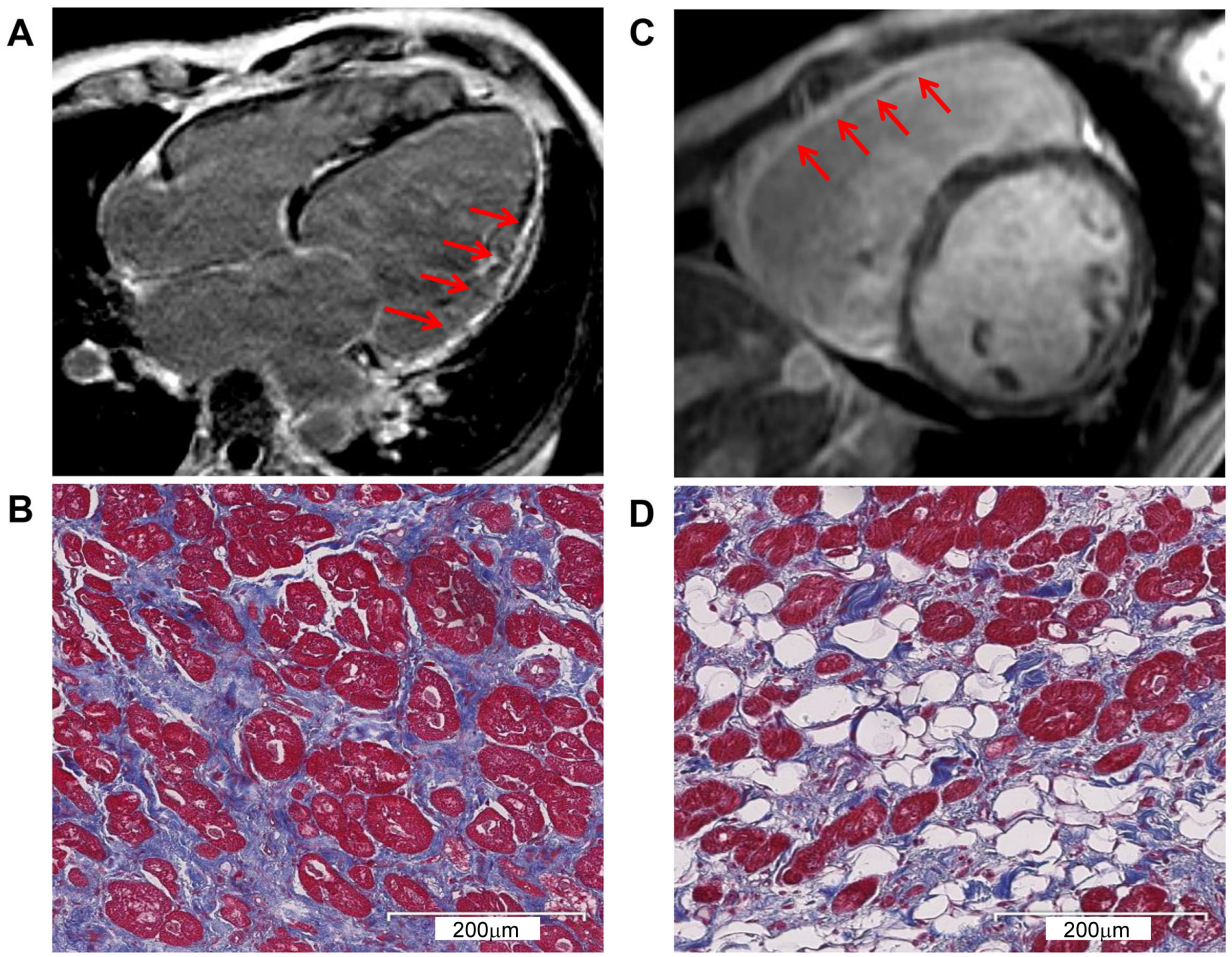
**CMR and pathological exam showed severe myocardial changes**. (A) CMR with LGE identified LV enlargement in P1. The arrows indicate severe myocardial involvement and LV fibrosis replacement. (B) Masson’s trichrome staining corresponding to (A) shows the LV interstitial fibrosis. Myocardium (red), fibrosis (blue), and fat (white). (C) CMR with LGE identified RV enlargement in P3. The arrows indicate severe myocardial involvement and RV fibrosis replacement. (D) Masson’s trichrome staining corresponding to (C) shows severe RV fibrofatty infiltration. Myocardium (red), fibrosis (blue), and fat (white). CMR, cardiac magnetic resonance; LGE, late gadolinium enhancement; LV, left ventricular; P1, proband 1; P3, proband 3; RV, right ventricular.

During follow-up all patients progressed to severe end-stage, i.e., irreversible heart dysfunction with an left ventricular ejection fraction (LVEF) <30%, and an enlarged left ventricular end-diastolic diameter (LVEDD) >6 cm. From the point of pregnancy, the six probands experienced different events, including syncope, implantable cardioverter-defibrillator (ICD) implantation, arrhythmia, and gradually progressed to HF. Cardiac magnetic resonance (CMR) with late gadolinium enhancement identified severe LV or right ventricular (RV) myocardial involvement. CMR images of two of the six probands are presented in Fig. 1A,C, respectively. Histological Masson’s trichrome staining of the myocardial tissue confirmed LV interstitial fibrosis and RV fibrofatty infiltration of proband 1 (P1) and P3, respectively (Fig. 1B,D).

### 3.2 Genetic Screening

Six women with PPCM underwent genetic testing. Pathogenic mutations were identified in four of the probands (P1, P2, P3 and P4) (Table 2). Both P2 and P3 had mutations in the gene encoding phospholamban (*PLN*) (c.36_38delAAG, p.Arg13del). P1 had a mutation in the gene encoding myosin binding protein C (*MYBPC3*). P4 carried a mutation in the gene encoding Filamin C (*FLNC*). All mutations were identified as pathogenic mutations according to the ACMG guidelines. No pathogenic mutation was identified in P5 or P6.

**Table 2.** **Mutations identified in the probands and their family members**.

Case	Gene	Nucleotide change	Amino acid change	Classification	Affected relatives carrier
P1	*MYBPC3*	c.C1408T	p.Arg470Trp	Pathogenic	II.3, II.9
P2	*PLN*	c.36_38delAAG	p.Arg13del	Pathogenic	II.6, II.7, III.7
P3	*PLN*	c.36_38delAAG	p.Arg13del	Pathogenic	II.2, II.1, III.3
P4	*FLNC*	c.2618delA	p.Glu873fs	Pathogenic	II.3

P, proband.

### 3.3 Family Screening 

**Fig. 2.**
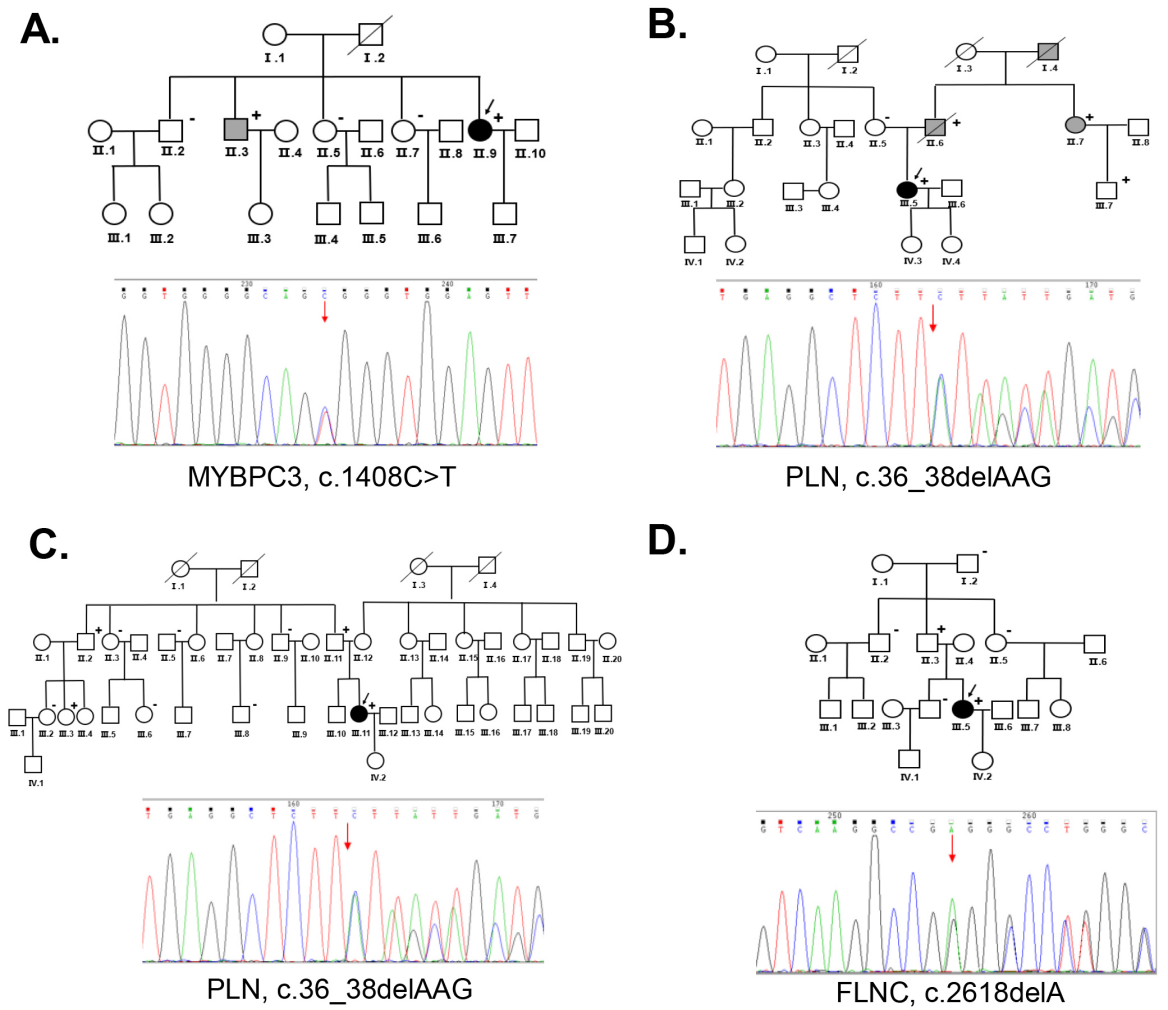
**The families of the probands with PPCM and genetic analysis**. (A) Pedigree of Family 1 (P1) and Sanger sequencing results confirming the *MYBPC3* gene mutation (c.1408C>T). (B) Pedigree of Family 2 (P2) and Sanger sequencing results confirming the *PLN* gene mutation (c.36_38delAAG). (C) Pedigree of Family 3 (P3) and Sanger sequencing results confirming the *PLN* gene mutation (c.36_38delAAG). (D) Pedigree of Family 4 (P4) and Sanger sequencing results confirming the *FLNC* gene mutation (c.2618delA). The squares represent males, and the circles represent females. The solid symbols indicate a clinical diagnosis of PPCM or DCM. Black indicates PPCM, and gray indicates DCM. The diagonal lines through the symbols indicate deceased individuals; the arrows indicate the proband in each family; and the presence or absence of a mutation is indicated by the + or – symbol, respectively. Below the family tree are the Sanger sequencing results with red arrows indicating the mutation sites. PPCM, peripartum cardiomyopathy; DCM, dilated cardiomyopathy; MYBPC3, myosin binding protein C; PLN, phospholamban; FLNC, filamin C.

P1 presented with peripheral edema and intermittent lower back pain during pregnancy. The condition worsened after giving birth, and the proband was treated with digoxin and spironolactone tablets. Over the next 8 years, she continued to experience dyspnea on exertion and orthopnea symptoms with regular medication for treatment. She developed palpitations and an ICD was inserted. Echocardiography showed an LVEF of 29% indicating severe myocardial involvement. She eventually developed end-stage HF. The genetic analysis showed that P1 carried a *MYBPC3* mutation (c.C1408T, p.Arg470Trp). Her brother (Ⅱ.3) was diagnosed with DCM and underwent heart transplantation, and he had the same* MYBPC3* mutation. Her other relatives (Ⅱ.2, Ⅱ.5, Ⅱ.7) were negative for the *MYBPC3* mutation during family screening (Fig. 2A). This mutation is believed to be pathogenic according to the ACMG guidelines.

P2 experienced syncope during the 3rd trimester of her second pregnancy. An ECG showed ventricular premature beats (VPBs) and T-wave changes. Echocardiography and ECG were regularly performed to monitor the heart function. The LVEDD increased to 6.83 cm and the LVEF declined to 28% during follow-up a period. The proband had palpitations without apparent triggering, and ventricular fibrillation was recorded by the ICD. A *PLN* mutation (c.36_38delAAG, p.Arg13del) was identified in this proband. The proband had a family history of DCM. Her aunt (Ⅱ.7) developed DCM after pregnancy and she had the same *PLN* mutation. A *PLN* mutation was also identified in her father who was diagnosed with DCM (Ⅱ.6). The *PLN* mutation was segregated to family members with DCM (Fig. 2B).

P3 showed symptoms of paroxysmal chest tightness, dyspnea, and bilateral lower-extremity edema at 34^+4^ weeks of pregnancy. Echocardiography demonstrated an LVEF of 19% with global cardiac enlargement, LVEDD of 6.2 cm, wall motion abnormality, and mitral annulus with moderate mitral regurgitation. She developed advanced HF and required heart transplantation 3 months after delivery. A *PLN* mutation (c.36_38delAAG, p.Arg13del) was identified in the proband. Her father (Ⅱ.11) had the same mutation but without indications of heart disease. Two of her relatives (Ⅱ.2, Ⅲ.3) also showed a positive result for this mutation. Her other family members (Ⅱ.3, Ⅱ.5, Ⅱ.9, Ⅲ.2, Ⅲ.6, and Ⅲ.8) tested negative for this mutation (Fig. 2C).

P4 presented with dyspnea 6 hours after delivery and was diagnosed with PPCM. She was discharged after treatment with an angiotensin-converting enzyme inhibitor and a β-blocker. She remained free from HF symptoms for the first 8 years, but her condition worsened after that time. At follow-up, the ICD was inserted to treat paroxysmal supraventricular tachycardia at the eleventh year. She then gradually developed end-stage HF. Her father (II.3) carried the same *FLNC* (c.2618del) mutation. Her other family members (Ⅰ.2, Ⅱ.2, Ⅱ.5, and Ⅲ.4) tested negative for this mutation (Fig. 2D).

### 3.4 Probands with Sporadic Disease

P5 was diagnosed with PPCM after presenting with chest tightness, dyspnea, fatigue, and bilateral lung congestion 3 days postpartum. Echocardiography demonstrated an LVEF of 25% with LV enlargement, pulmonary hypertension (59 mmHg), and mitral annulus with moderate mitral regurgitation. ECG showed VPBs (ectopic beats) and conduction block. This proband developed severe myocardial involvement, left heart dysfunction, and pulmonary hypertension within 1 year. No pathogenic mutation was identified.

P6 presented no obvious abnormality during pregnancy. However, she experienced chest tightness, dyspnea, and syncope 3 months postpartum and was diagnosed with PPCM. Digoxin and metoprolol were used to reverse ventricular remodeling. Initially, she was discharged after her condition improved. Echocardiograms were regularly performed to monitor disease development. In the second year, an ECG showed non-sustained ventricular tachycardia and atrial fibrillation, and echocardiography revealed decreased left heart function and pulmonary hypertension with an LVEF of 24%. She finally progressed to severe cardiomyopathy and HF in the fourth year after she suddenly lost consciousness. No pathogenic mutation was identified.

### 3.5 Penetrance Analysis

**Fig. 3.**
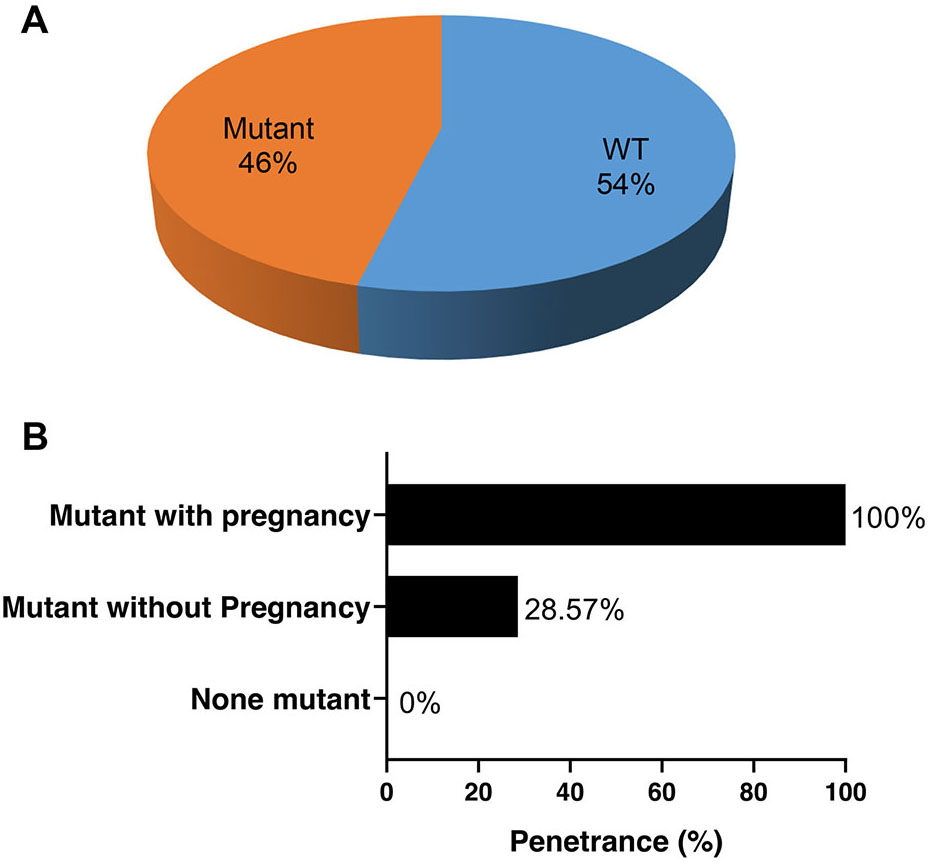
**Penetrance of heart disease in the family members of the 4 probands with pathogenic mutations**. (A) The proportion of mutation carriers among the family members who underwent screening. (B) Penetrance of the mutations with and without pregnancy. WT, without mutant.

We screened the clinical condition and performed genetic testing on their family members of the four probands with pathogenic mutations. Among the 26 family members that underwent genetic testing, 14 (54%) were identified as genotype-negative, while 12 were identified as genotype-positive (46%) (Fig. 3A). Among the women who tested positive for the mutations, symptoms were observed in all pregnancies, while only 28.57% of the mutation carriers without pregnancies exhibited symptoms at the time of screening. This suggests that pregnancy may facilitate the penetrance of these gene mutations (Fig. 3B).

## 4. Discussion

PPCM is a severe condition that is observed in pregnant women throughout the world. Although PPCM is reported to have a high rate of recovery of ventricular function [28], around 10%–20% of patients still face severe cardiomyopathy and experience major adverse cardiovascular events [29, 30].

In the present study, all of the probands had an LVEF of <30% and an LVEDD of >6.0 cm with severe LV dysfunction. They gradually progressed to severe HF requiring heart transplantation. At least 10% of patients with PPCM who do not recover significantly with medical treatment will require heart transplantation [21]. The similar clinical characteristics of poor LVEF and LV enlargement that were shared by the 6 probands appeared to predict their poor outcomes. McNamara previously reported a similar finding that no women with an initial LVEF of <30% and an LVEDD of >6.0 cm recovered normal systolic function [21]. The presence of an underlying genetic mutation or a positive family history of inherited cardiomyopathy, especially DCM, may also contribute to the low recovery rate in our PPCM cohort.

PPCM may be an initial manifestation of FDCM, which shares similar clinical features and genetic mutations that may contribute to its pathogenesis [13]. In this study, we screened the genetic mutations of the 6 probands with PPCM and their family members to evaluate the contribution of these variants to PPCM. In this report, PPCM was diagnosed in 6 probands and we were able to identify that four of them had pathogenic mutations associated with DCM, HF, or arrhythmia. For P1, the *MYBPC3* mutation was also identified in her brother who was diagnosed with DCM. The *MYBPC3* mutation, which can be associated with cardiac events such as HF, has previously been identified as a causative mutation in DCM [31]. This mutation may be the fundamental risk factor for P1 developing PPCM during pregnancy. In the families of P2 and P3, the same *PLN *mutation (c.36_38delAAG, p.Arg13del) was identified as pathogenic. *PLN* encodes phospholamban, which is a reversible inhibitor of cardiac sarcoplasmic reticulum calcium ATPase isoform 2a (*SERCA2a*), and mutations in *PLN* have previously been identified in patients with DCM, PPCM, and severe HF [32, 33]. Notably, in the family of P2, the aunt (II.7) was diagnosed with DCM after pregnancy, and she had the same *PLN *mutation as the proband, which indicates that pregnancy may accelerate the penetrance of this mutation. Her aunt’s son (Ⅲ.7) also had the same mutation, but he did not show any symptoms of heart disease at the time of screening. This may be because of the gene’s penetrance during pregnancy. In the family of P3, a total of 10 members were tested for the mutation. No first-degree relatives (with or without the mutation) showed symptoms of heart disease. Given the large size of this family and the fact that only the proband developed heart disease after pregnancy, we suggest that pregnancy may accelerate disease manifestations in women carrying cardiomyopathy-related mutations. In the family of P4, the *FLNC* mutation (c.2618delA, p.Glu873fs) was identified in the proband (Ⅲ.5) and her father (Ⅱ.3). *FLNC* has been implicated in inherited forms of cardiomyopathy and as the cause of DCM with life-threatening ventricular arrhythmia [34, 35]. The *FLCN* mutation explained the arrhythmia phenotype shown by the proband. For the families of P5 and P6, no pathogenic mutation was identified. In the future, whole-genome sequencing may be useful to identify mutations in these two families.

Pregnancy is associated with structural, physiological and hormonal changes, including an increase in blood volume, cardiac output related to increased heart rate and physiological upregulation of prolactin [17, 36]. Cardiac remodeling further leads to a substantial increase in LV mass and enhanced angiogenesis [37]. These changes occur to meet the needs of the mother and fetus. Shibuya *et al*. [38] reported a PPCM woman with low prolactin blood levels who recovered from PPCM with anti-prolactin therapy. These non-genetic factors that are inherent in pregnancy may result in gene mutation penetrance and entail a risk of developing HF or arrhythmias in women who carry a cardiomyopathy-related genetic mutation [39]. In this study, the penetrance of any symptoms of heart disease was 100% in mutation carriers during pregnancy. However, not all individuals with mutations showed symptoms; the penetrance of mutation carriers without pregnancy was only 28.57%. This indicates that pregnancy may be a strong factor for the penetrance of gene mutations.

In general, typical patients with PPCM demonstrate a benign course with recovery of LV function. However, it has also been reported that the outcomes of patients with PPCM in families with DCM appear to be worse with a lower chance of recovery compared with PPCM patients without family history [20]. A subset of cases of PPCM have been associated with the familial spectrum of DCM [40]. Therefore, for families with a history of DCM, genetic counselling and screening are recommended for first-degree female relatives with potential reproductive risk [13]. Further, women with a first-degree relative who has been diagnosed with DCM should be followed and monitored during pregnancy. These families may benefit from genetic counselling; therefore, careful inquiry about family history should be performed for patients with PPCM. The correlation between gene mutations and PPCM may also facilitate the implementation of genetic screening for PPCM, particularly in high-risk women with family members affected by PPCM or DCM. Detecting these mutations would greatly improve the capability to identify women who are at risk of developing PPCM. Early monitoring of such patients and risk stratification could improve the survival rate of patients with PPCM.

There were several limitations in our study. First, this is a single-center retrospective study, which may restrict the generalizability of our findings to broader populations. Multi-center studies with prospective designs would be instrumental in confirming and extending the applicability of our findings across diverse populations. Additionally, the relatively small sample size of PPCM patients may diminish the statistical power. Last, it is crucial to acknowledge that PPCM likely involves a complex interplay of genetic predisposition and environmental, social, and hormonal factors. While our study identified genes associated with DCM in PPCM cases, it is essential to recognize the potential limitations in definitively categorizing these genes as exclusive causative factors for PPCM.

## 5. Conclusions

In conclusion, we reported 6 probands with PPCM who experienced severe outcomes. They all shared similar clinical manifestations, which reflected the worsening condition of the heart. Our study also underscored the genetic predisposition for PPCM. Women with a positive genetic background of DCM or a family history of DCM may be susceptible to poor recovery from cardiac dysfunction and HF. Long-term monitoring may be essential for patients with PPCM.

## Data Availability

Datasets in this study are available from the corresponding author, upon reasonable requests.

## Abbreviations

PPCM, peripartum cardiomyopathy; LV, left ventricular; HF, heart failure; DCM, dilated cardiomyopathy; LVEF, left ventricular ejection fraction; LVEDD, left ventricular end-diastolic diameter.

## Supplementary Material

Supplementary material associated with this article can be found, in the online version, at https://doi.org/10.31083/j.rcm2511399.
