# Supplementary material for: Gene Analysis of Four Families with Severe Peripartum Cardiomyopathy Reveals Known Gene Mutations and Supports the Recent Call for Screening
Source: Rev Cardiovasc Med. 2024 Nov 14;25(11):399. doi: 10.31083/j.rcm2511399 (PMC11607517; doi:10.31083/j.rcm2511399)
Supplement: Supplementary file 1 [file 2153-8174-25-11-399-s1.docx]

Supplementary Material

Supplementary Table 1. Panel genes.

| Gene | Gene Name | Transcript | #exons |
| --- | --- | --- | --- |
| ABCC9 | ATP-binding cassette, subfamily C | NM_005691.2 NM_020297.2 | 38  38 |
| ABCG5 | ATP-binding cassette, subfamily G, member 5 | NM_022436.2 | 13 |
| ACADVL | Acyl-CoA dehydrogenase, very long chain | NM_000018.2 | 20 |
| ACTA1 | Actin, alpha 1, skeletal muscle | NM_001100.3 | 7 |
| ACTA2 | Actin, alpha 2, smooth muscle, aorta | NM_001613.2 NM_001141945.1 | 9  9 |
| ACTC1 | Actin, alpha | NM_005159.4 | 7 |
| ACTN2 | Alpha actinin | NM_001103.2 | 21 |
| ADRA1A | Alpha-1A-adrenergic receptor | NM_000680.2 NM_033304.2 NM_033303.3 | 2  3  3 |
| ADRA2A | Alpha-2A-adrenergic receptor | NM_000681.3 | 1 |
| ADRA2B | Alpha-2B-adrenergic receptor | NM_000682.5 | 1 |
| ADRA2C | Alpha-2C-adrenergic receptor | NM_000683.3 | 1 |
| ADRB1 | Beta-1-adrenergic recepter | NM_000684.2 | 1 |
| ADRB2 | Beta-2-adrenergic recepter | NM_000024.5 | 1 |
| AGT | Angiotensinogen | NM_000029.3 | 5 |
| AKAP6 | A-Kinase Anchor protein | NM_004274.4 | 14 |
| AKAP9 | A kinase (PRKA) anchor protein 9 | NM_005751.4 | 50 |
| ALG6 | Asparagine-linked glycosylation 6, alpha- 1,3-glucosyltransferase homolog | NM_013339.3 | 15 |
| ANK2 | Ankyrin 2 | NM_001148.4 | 46 |
| ANKRD1 | Ankyrin repeat domain- containing protein 1 | NM_014391.2 | 9 |
| AP3B1 | Adaptor-related protein complex 3, beta 1 subunit | NM_003664.3 | 27 |
| APLNR | Apelin recepter | NM_005161.4 | 2 |
| AQP4 | Aquaporin 4 | NM_001650.4 | 5 |
| ARL6 | ADP-ribosylation factor- like 6 | NM_032146.3 NM_177976.1 | 9  9 |
